# Implementing infection prevention and control capacity building strategies within the context of Ebola outbreak in a “Hard-to-Reach” area of Liberia

**DOI:** 10.11604/pamj.2018.31.107.15517

**Published:** 2018-10-12

**Authors:** Michael Ogbonnaya Oji, Mesfin Haile, April Baller, Nathalie Tremblay, Nuha Mahmoud, Alex Gasasira, Victor Ladele, Catherine Cooper, Francis Ndivo Kateh, Tolbert Nyenswah, Peter Nsubuga

**Affiliations:** 1 Infection Prevention and Control, Health Security and Emergencies Department, World Health Organization, Monrovia, Liberia; 2 Department of Quality Management Unit Services, Ministry of Health and Social Welfare, Monrovia, Liberia; 3 Public Health Unit, Global Public Health Solutions, Atlanta GA, USA

**Keywords:** Infection prevention and control, hard-to-reach areas, capacity building, Ebola virus disease

## Abstract

**Introduction:**

In August 2014, WHO declared that Ebola outbreak ravaging West Africa including Liberia had become a Public Health Emergency of International Concern (PHEIC). Infection prevention and control (IPC) among healthcare workers was pivotal in reducing healthcare worker infection and containing the recent EVD outbreak. Hard to reach areas (HTRA) presents peculiar challenges in public health emergencies. We present the result of IPC capacity building strategies deployed in Gbarpolu County: an HTRA of Liberia.

**Methods:**

Between April to October 2015, we conducted IPC training and mentorship at the county, district and facility levels in a selected HTRA of Liberia using the keep Safe, Keep Serving manual and the WHO core components of infection control. Serial follow-up assessments and mentoring using the Liberian Minimum standard tool for safe care in Liberian health facilities (MST) were done.

**Results:**

180 (100%) facility based healthcare workers were trained: including 59 clinicians (32%) and 121 (67%) non-clinicians. 100% of the healthcare workers in four selected very HTRAs were trained and underwent facility based-mentorship. Compliance with IPC practice increased: the MST score increased from 75% to 90% and for the MST score for waste management and isolation increased 60% to 87%.

**Conclusion:**

Strengthening the capacity of healthcare workers for IPC was instrumental for containing the EVD epidemic but also critical for routine safe and quality services. A culture of IPC among healthcare workers in HTRA can be implemented through capacity building and training.

## Introduction

The Ebola Virus Disease (EVD) outbreak which started in the forests of Guinea in Dec 2013, was the worst acute public health crisis in the last 50 years and eventually became a public health emergency of international concern [1-3]. Many West African countries including Liberia, Guinea, Sierra Leone, Senegal, and Nigeria were affected. The Liberian EVD outbreak began in March 2013 in the forest regions of Lofa County and affected several parts of the country including remote and rural areas. The Ebola Virus Disease outbreak in West Africa, was associated with 28,616 confirmed, probable and suspected cases reported in Guinea, Liberia, Sierra Leone with 11,310 deaths; Liberia had witnessed a total of 10,675 confirmed cases, 4,809 deaths including 378 health worker infections, 192 deaths (approximately 8.07%) of Liberian Health workers [4-6]. Urgent public health interventions including robust infection prevention and control (IPC) measures were necessary to stop transmission as well as treat those affected. Public health interventions, especially in large emergency operations like the EVD outbreak, are often constrained by geographical, physical, and other barriers, especially in very remote, isolated and hard-to-reach areas. Hard-to-reach areas are those places with geographical, physical, communication, security, social, and economic barriers that make them receive a level of public service that is relatively inequitable and below the national benchmark [7-9]. Gbarpolu County is a land-locked area located in western Liberia; it shares a border directly with Sierra Leone, with Lofa County (which shares a border with Guinea), and Grand Cape Mount County. These border connections have implications for epidemiology, disease prevention, and control. Gbarpolu has health facility density index of 1.45 per 10,000 population (which is one of the lowest in Liberia) and a population of 96,446 [10]. The County has five health districts and six political districts and has dense forests, poor basic infrastructure, and a poor road network, with no paved roads and the majority of the roads, are tertiary roads or trails [11]. Only 36% of the population lives within 5 kilometres of a health facility, which is the lowest in Liberia [10]. Most villages and towns are separated by dense forests with track roads and wooden bridges the majority of which are in a grave state of disrepair. During the rainy season, many of the roads frequently become unpassable because of complete disconnection, destroyed bridges and sometimes by rising water levels. Some areas are encircled by rivers making access difficult during the rainy season. This terrain often discourages many partners and health care workers to work in the County. During the March 2014 to May 2015 EVD epidemic, the County had 24 confirmed cases and 16 deaths (67%). With frequent traffic of human and animals between Sierra Leone and Gbarpolu, transmission and re-introduction of the infection from that axis remained a constant threat. Health workers are not only vulnerable but can be part of the transmission chain as well as health facility contacts [12]. Lack of relevant training, knowledge and practice in IPC was a common gap among health workers in the countries affected by the outbreak [13]. Knowledge and practice of IPC increase the confidence of healthcare workers and reduces the fear and myths associated with the EVD epidemic. It was important therefore during and after the EVD to build the IPC capacity of the healthcare workers in the country as well as increase community awareness of basic IPC practices and its role in self-protection and disease control. We highlight the combination strategies that were used to build local capacity and improve IPC practice in the health facilities and among the communities in Gbarpolu County. It is hoped that this might serve as a useful model for implementing IPC capacity building in other hard to reach areas.

## Methods

We identified four key strategies and four levels of interventions as necessary to build IPC capacity in Gbarpolu County. The four levels were the county, district, facility, and the community levels. The key strategies we identified and implemented were to build IPC capacity through training, mentorship, improvement in supply chain management and enhanced health facility-community engagement. We focused on the County IPC focal person and selected members of the County Health Team (CHT) who were clinicians and had some oversight functions to the health facilities in the County. We implemented these interventions with the CHT and other partners working in the County. We ensured and improved coordination through the weekly, monthly, and quarterly County IPC review meetings.

**Process, training, and materials:** First, we conducted a baseline county facility assessment at some selected facilities based on proximity and accessibility. We assessed six facilities: five clinics and the hospital in three out of the five health districts. The baseline facility assessment indicated gaps in IPC practice and compliance. Secondly, we developed a training programme and implemented it at the county, district, and facility levels. Training consisted of didactic lectures with PowerPoint presentations, group work, skill demonstration, e.g, use of personal protective equipment (PPE) and hand washing techniques as well as direct health worker engagement and mentoring. The training materials were the Keep Safe, Keep Serving Infection Control manual (KSKS), December 2014 version [14, 15], the WHO eight core components of IPC 2011 version [16] and the Liberian Ministry of Health's Health Facilities Minimum Standards for Safe Care Provision in the Context of Ebola document also known as the “Minimum Standards Tool” (MST) [17]. All were documents approved by the Liberian Ministry of Health. The MST is a five-page survey based on the eight core components of IPC. It is used to assess various IPC components including administrative information, standard operating procedure (SOP) availability, waste management, equipment and supplies, personnel staffing and training, triage, isolation and other miscellaneous items ((e.g,availability of electricity). For the county and district level training, we categorised selected participants into two groups: the first group consisted of the Officers-in-Charge (OIC) of clinics (usually a nurse or physician assistant) and IPC focal persons. The second group included the County IPC focal person and selected members of the CHT and the District Health Officers (DHOs). The first group was trained on the Keep Safe Keep serving (KSKS) infection control package, the eight core components of IPC, and general awareness of the MST while the second group was trained in the use of the MST tool for assessment in addition to all the training that was given to the first group. After the training, the participants returned to their facilities and conducted training for healthcare workers in their facilities within the following 2 weeks. After that, we commenced monthly follow-up supportive supervision and assessment visits by a combined IPC team of WHO, CHT, and other partners. The visits were used to assess healthcare workers' and facility's compliance with the MST and as an opportunity for healthcare worker engagement, mentoring, clinical process tracking, and building the confidence of healthcare workers for IPC compliance. In very hard-to-reach areas for which cumbersome logistics impeded routine visits, we conducted on-site facility training and mentoring which lasted about 2 to 5 days depending on pre-MST assessment findings and identified gaps. The assessment involved a visit to the facility, which was often unannounced, by assessors who were pre-trained on the use of the MST tool. Healthcare workers were interviewed and observations made based on the survey items on the MST. For each item, the assessor ticked either 'Yes' or 'No' in the space provided on the survey tool document. Feedback was given to the healthcare workers immediately on any issues that needed correcting and on-site spot mentorship given. Gaps that were not immediately correctable were noted and referred to the County IPC committee, the CHT or the National IPC task force as was appropriate. We encouraged and conducted community engagement as part of the mentoring process. This enhanced facility-community collaboration and also helped to deal with identified gaps that were correctable. We held meetings with relevant community leaders and other stakeholders at a pre-arranged venue, which was usually at the 'palaver' or town hall. We explained and where necessary used job aids to emphasise the importance of triage control and IPC for individual, family and community benefits and why healthcare workers have to wear PPE. We ensured that community engagement sessions were an interactive session with opportunities for clarifications, questions, and answers.

**IPC supply chain management:** Because of the nature of the terrain, another partner John Snow Incorporated (JSI) was contracted to conduct the 'last mile' distribution of IPC materials in the County. We also checked IPC supply distribution in the facilities. Specific materials lacking were identified and communicated to JSI directly or during the county IPC committee meetings. We had joint discussions with JSI and planned for special distribution to very hard-to-reach areas. For example, instead of monthly distribution, some received 3-month supply. JSI also incorporated supportive IPC supervision and mentoring as part of this supply chain management.

**Data collection and analysis:** Data were collected from the assessment of the facility compliance with IPC using the MST tool. For analysis, each score on the MST was assigned a numerical value: No= 0, Yes=1. Data analysis was done with Microsoft Excel version 2010.

## Results

**Figure 1:**
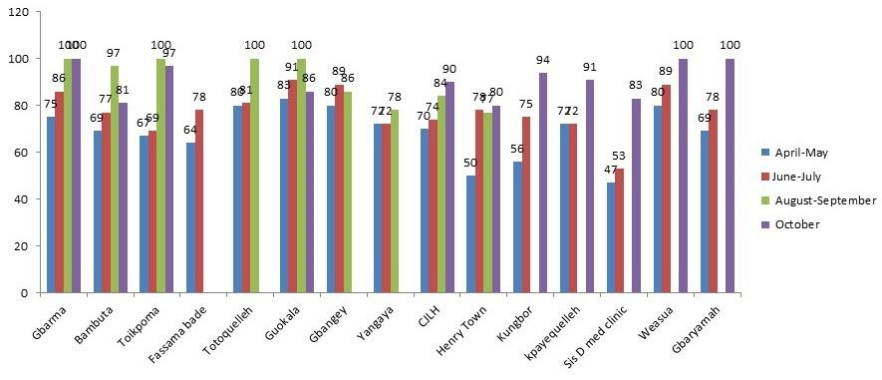
Percentage compliance with MST among health facilities in Gbarpolu county (April to October 2015)

**Figure 2:**
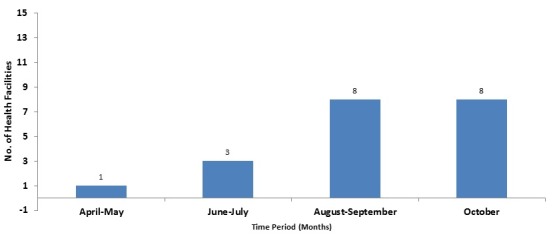
Cumulative number of health facilities with on-going or completed triage area (Facility-community-Partner- collaboration) in Gbarpolu County, April to October 2015

**Figure 3:**
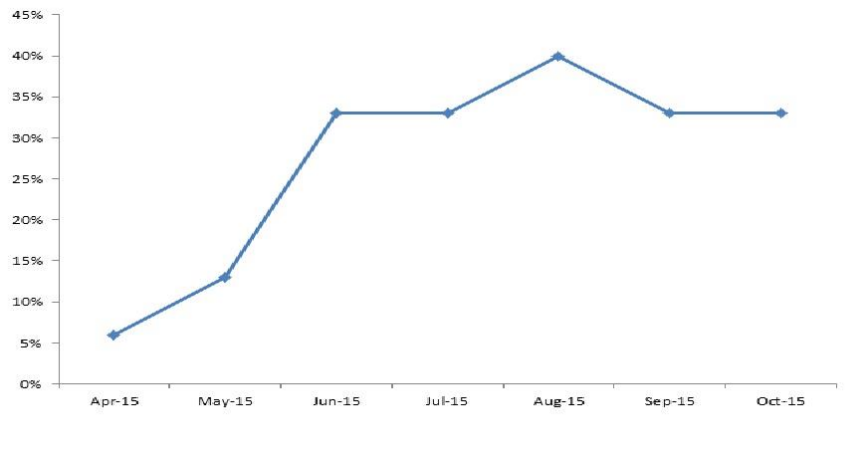
Percentage of assessed facilities in Gbarpolu County that have isolation place -for patients waiting urgent transfer (April to October 2015)

**Figure 4:**
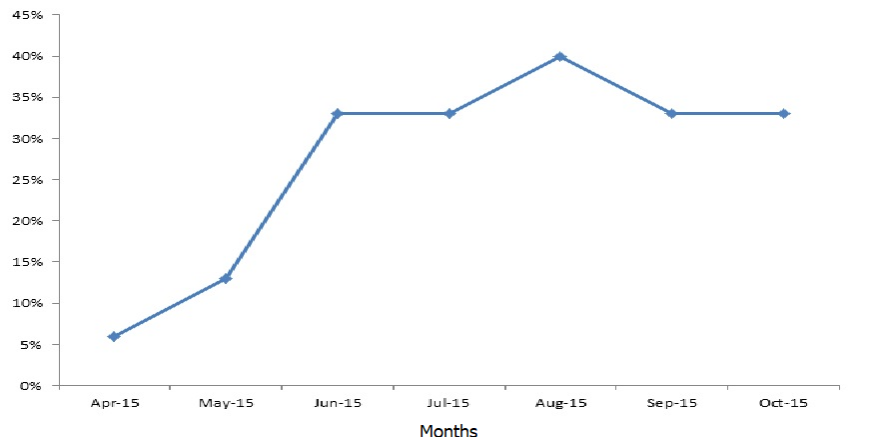
Percentage of health facilities in Gbarpolu County with waste management system according to Ministry of Health standard (April to October 2015)

There were 15 health facilities, (14 clinics, and one hospital). Of the 14 clinics, 13 (93%) are public and one private. A total of 180 facility-based health workers participated in the training at county and district (45 persons) and facility level (135 persons). All the facility based staff were trained. They were 118 males and 52 females; the male to female ratio was 2:1. The trainees included 59 clinicians-physician assistants, nurses and midwives (32.8%); while non-clinicians were 121 (67.2%), giving a clinician to a non-clinician ratio of 1:3. Non-clinicians included 27(15.0%) nurse aides, 19(10.6%) dispensers and 19 (10%) registrars, while cleaners and security workers were 20(11.1%), (Table 1). Among the very hard-to-reach areas, Belle Fassama and Kungbor clinics had 10 healthcare workers each; Kpayequelleh and Weasua clinics had eight and seven respectively. All the clinic based staffs in the very hard to reach areas were trained (Table 2). Compliance with MST increased from 75% in April-May 2015 to more than 80% in June-July for Gbarma and Guokala clinics and 90% in October 2015. There was a drop in compliance in Bambuta and Guokala clinics in October compliance from almost 100% to 80% in both places. The compliance rate for Kungbor, Kpayequelleh and Weasua clinics increased from 60%, 70% and 80% (respectively) to approximately 100% in all three districts. Chief Jallah Leone Hospital (CJLH) maintained a consistent increase in percentage compliance (Figure 1). Within the period under consideration, the number of facilities with health facility clinic-community initiated triage increased from one in April-May to eight in August-September. This represented more than 50% of the facilities in the County (Figure 2). The number and percentage of health facilities with identifiable isolation place increased from one (6%) in April to approximately (six) 40% in August 2015 with a reduction to (five) 30% in September-October (Figure 3). The percentage of compliance with standard waste and sharps management according to the Ministry of Health protocol increased from 60% in April, to 70% in May and peaked at about 87% in June. There was a reduction in September (60%) and increased again in October to 70% (Figure 4).

**Table 1:** Distribution of healthcare workers trained by professional category in Gbarpolu County, April to October, 2015

Non-Clinicians (NC)	No.	%
Pharmacists	1	0.6
Environmental health technicians (EHT)	5	2.8
Lab Technician	2	1.1
Nurse Aide	27	15.0
Lab Aide	8	4.4
Dispensers	19	10.6
Registrars	19	10.6
Security	20	11.1
Cleaner	20	11.1
Total	121	67.3
**Clinicians (C)**		
Nurses	30	16.7
Midwives	20	11.1
Physician assistants	7	3.8
Physicians	2	1.11
Total	59	32.7
Combined Total (C and NC)	180	100

**Table 2:** Percentage of HCWs that had facility based training and mentoring in very HTRA in Gbarpolu county (April to October 2015)

District	Health facility	No. of healthcare workers (HCWs) trained			
Belle	Belle fassama community clinic	3	7	10	100
Bopolu	Weasua community clinic	3	4	7	100
Kumgba	Kungbor community clinic	3	7	10	100
Guolala	Kpayequelleh community clinic	3	5	8	100

## Discussion

Between April to October 2015, we found that a combination of county, district, and facility level training of healthcare workers, re-enforced by regular supportive supervisory assessment and mentoring visits as well as improved supply chain management significantly increased the capacity of the health care workers and health facilities in Gbarpolu county to implement appropriate IPC practices during the EVD outbreak. Training involving knowledge and skill transfer is a key component of capacity building for all health workers including security staffs and cleaners; all of whom are vulnerable [12]. Multi-level training (county, district, and facility) and facility assessment visits provided an opportunity for re-enforcement and mentoring. The strategy of country-level capacity building enabled the county clinical staffs to support IPC practices as part of their routine supervisory visits. Other authors like Rosenthal *et al* [18] in Argentina in 2003 and Burke *et al* in their work in the USA,2006 [19] noted that training incorporating participants' engagement, education, mentoring and feedback improved health workers compliance with IPC and safety practices. This is especially relevant in Liberia and West African sub-region where EVD and IPC knowledge and capacity has been deficient [1, 5, 20-24]. One of the evidence of strengthened health workers' capacity for IPC was improved compliance with IPC standards and protocols as assessed using the MST. There was a consistent increase in percentage MST compliance for all the assessed facilities from April-May to August-September period. This reflected improvement in compliance with standards of IPC standards and practice. Furthermore, compliance of health workers at the facilities with regard to isolation and waste management increased rapidly following the training and mentoring. Unfortunately, this was followed by a decline in August both in isolation and waste management. This coincided with the period of containment of the second EVD outbreak at Magibi and the anticipation that Liberia will be declared free in September 2015. Many health workers felt that Ebola has gone and were no longer strict on compliance or vigilance for IPC practices. Barbarossa *et al.* [25] in their projections of the 2014/15 Liberian EVD outbreak have expressed this concern of premature drop in compliance. In response, we conducted focused facility visits with resultant improvements in the September assessment. The increased number of facilities with locally initiated triage construction (Figure 3) may have reflected the impact of improved clinic and community awareness of IPC as well as ownership and collaboration to support IPC. This was one of the key messages during facility mentorship and community engagement. Community engagement and initiatives were one of the key interventions in containing the Masindi 2000 EVD outbreak in Uganda [20]. This is similar to our experience in Gbarpolu; community engagement resulted in communities taking ownership and support initiative for the construction of triage structure and fencing. Even though the focus of this paper is on capacity building for health workers, we note and acknowledge other partners who were involved in “community IPC” (e.g, distribution of household hygiene kits and re-enforcement of IPC and hygiene messages at family and community levels). These may have also influenced the positive attitude of the community, though this may be difficult to measure. We recognize some limitations of this study. The use of MST as an assessment tool tended towards assessment of more structural than process issues and secondly the problem of inter-observer error. To reduce the error from these: assessments were done by IPC focal persons as part of a joint assessment team (at least two persons), engagement with health workers while at work and mentoring sessions aimed to augment the process deficiencies in the MST.

## Conclusion

Appropriate training, mentoring, health worker engagements and facility assessments resulted in improved capacity for IPC compliance among health workers in Gbarpolu County. We recommend the capacity building of healthcare workers in hard-to-reach areas in such critical areas as IPC both as part of epidemic response preparedness and routine healthcare because when there is an outbreak, external help may be delayed. Secondly, in planning for public health interventions, emergency or routine, special, location-specific considerations need to be given hard to reach areas to ensure an improved outcome. Thirdly a separate, efficient supply chain management that puts into consideration the peculiarities of the terrain is critical for the success of healthcare programmes in HTRAs. Fourthly, government intervention is necessary to address the issues that make certain areas hard to reach. For Gbarpolu County, this is mainly physical and geographical barriers which could be overcome by improved access roads and network coverage.

### What is known about this topic

- Lack of knowledge and compliance with infection prevention and control principles and practices exposes health workers to increased risk and vulnerability to health care-associated infections often with dangerous consequences;
- Hard to reach areas are often under-served in public health emergencies interventions.

### What this study adds

- Building the capacity of health workers working in hard to reach but vulnerable areas is a key component for preparedness in communicable disease outbreaks and public health emergencies;
- Mentorship and capacity building promotes individual, and community ownership and this is important in preparing for and containing public health emergencies like Ebola Outbreak.

## Competing interests

The author declare no competing interests.
